# Understanding the Relationship between Environmental Arsenic and Prostate Cancer Aggressiveness among African-American and European-American Men in North Carolina

**DOI:** 10.3390/ijerph17228364

**Published:** 2020-11-12

**Authors:** Humberto Parada, Tianying Wu, Rebecca C. Fry, Laura Farnan, Gary J. Smith, James L. Mohler, Jeannette T. Bensen

**Affiliations:** 1 Division of Epidemiology & Biostatistics, School of Public Health, San Diego State University, San Diego, CA 92182, USA; tianying.wu@sdsu.edu; 2 Moores Cancer Center, University of California San Diego, La Jolla, CA 92093, USA; 3 Environmental Sciences and Engineering, Gillings School of Global Public Health, University of North Carolina at Chapel Hill, Chapel Hill, NC 27599, USA; rfry@email.unc.edu; 4 Department of Epidemiology, Gillings School of Global Public Health, University of North Carolina at Chapel Hill, Chapel Hill, NC 27599, USA; laura_farnan@med.unc.edu; 5 Department of Urology, Roswell Park Comprehensive Cancer Center, Buffalo, NY 14203, USA; gary.smith@roswellpark.org (G.J.S.); james.mohler@roswellpark.org (J.L.M.); 6 Lineberger Comprehensive Cancer Center, University of North Carolina at Chapel Hill, Chapel Hill, NC 27514, USA; jeannette_bensen@med.unc.edu

**Keywords:** prostate, cancer, environmental arsenic, inorganic arsenic

## Abstract

High-level exposure to arsenic, a known carcinogen and endocrine disruptor, is associated with prostate cancer (PCa) mortality. Whether low-level exposure is associated with PCa aggressiveness remains unknown. We examined the association between urinary arsenic and PCa aggressiveness among men in North Carolina. This cross-sectional study included 463 African-American and 491 European-American men with newly diagnosed, histologically confirmed prostate adenocarcinoma. PCa aggressiveness was defined as low aggressive (Gleason score < 7, stage = cT1–cT2, and PSA < 10 ng/mL) versus intermediate/high aggressive (all other cases). Total arsenic and arsenical species (inorganic arsenic (iAs^III^ + iAs^V^), arsenobetaine, monomethyl arsenic, and dimethyl arsenic)) and specific gravity were measured in spot urine samples obtained an average of 23.7 weeks after diagnosis. Multivariable logistic regression was used to estimate the covariate-adjusted odds ratios (ORs) and 95% confidence intervals (CIs) for PCa aggressiveness in association with arsenic tertiles/quantiles overall and by race. The highest (vs. lowest) tertile of total arsenic was associated with PCa aggressiveness ORs of 1.77 (95% CI = 1.05–2.98) among European-American men, and 0.94 (95% CI = 0.57–1.56) among African-American men (*P*_Interaction_ = 0.04). In contrast, total arsenic and arsenical species were not associated with PCa aggressiveness in unstratified models. Low-level arsenic exposure may be associated with PCa aggressiveness among European-Americans, but not among African-Americans.

## 1. Introduction

Prostate cancer (PCa) is the most frequently diagnosed non-skin cancer and the second leading cause of cancer-related death among men in the United States (US) [1]. In 2020, an estimated 190,000 men will be diagnosed with PCa and 33,000 men will die from the disease [1]. PCa incidence rates declined in the last decade [2], in part due to decreased screening since the US Preventive Services Task Force recommended against the use of prostate-specific antigen (PSA) testing in 2008 [3] and 2012 [4], but racial disparities in PCa persist. African-American men have the highest PCa burden of any racial or ethnic group [5] and are more likely to be diagnosed at younger ages [6], present with a more aggressive form of the disease [7], and have greater mortality rates [5] than European-American men. Few modifiable risk factors have been identified [8,9] despite substantial research aimed at understanding the etiology of PCa and the determinants of these disparities [10]. However, accumulating epidemiologic and laboratory evidence indicates that arsenic may play a role in the pathogenesis of PCa among highly and chronically exposed men [11]. Whether low-level chronic arsenic exposure is associated with PCa aggressiveness remains unknown.

Arsenic is a metalloid that occurs naturally in different oxidation states and as toxic inorganic (e.g., arsenate (iAs^V^) and arsenite (iAs^III^)) and non-toxic organic (e.g., arsenobetaine (Ab)) compounds [12]. Among the general US population, exposure to iAs, an established carcinogen [13] and endocrine disruptor [14], occurs primarily through drinking water [15]. In North Carolina (NC), iAs is naturally occurring originating from the bedrock of the slate belt and private drinking wells have been found to have iAs levels that exceed the Environmental Protection Agency drinking water standard of 10 ppb [16]. This is of public health concern given that about 2.4 million North Carolinians rely on groundwater as their primary drinking water source [17].

Once ingested, iAs is absorbed from the gastrointestinal tract and distributed throughout the body where it may undergo biotransformation in the liver and other tissues by methylation [18]. Methylated intermediates including monomethyl arsenic (MMA) and dimethyl arsenic (DMA) have potential carcinogenic and tumor-promoting effects [19], although iAs and methylated metabolites are excreted in urine within 4–5 days and have relatively low rates of bioaccumulation [20]. Urine is, therefore, the most common and reliable biological matrix for the assessment of arsenic exposure in epidemiologic studies.

In this study, we examined the associations between urinary levels of total arsenic as well as the arsenical species iAs, DMA, MMA, and PCa aggressiveness overall by race among NC men who participated in a population-based study. We hypothesized that PCa aggressiveness would be associated with greater urinary As levels given the tumor-promoting effects of arsenic metabolites and that African-American men would have higher arsenic levels since they have been historically excluded from regulatory water services and are, therefore, more likely to rely on individual water sources such as wells [21], and thus have a greater proportion of aggressive prostate cancer.

## 2. Materials and Methods

### 2.1. Study Population

This cross-sectional study used resources from the North Carolina-Louisiana Prostate Cancer Project (PCaP), a population-based cohort study designed to examine racial differences in PCa outcomes between African-American and European-American men [22]. This study focused on NC men enrolled in PCaP. From 2004 to 2007, 472 African-American men and 501 European-American men from NC with a new diagnosis of histologically confirmed prostate adenocarcinoma between the ages of 40 and 79 years at diagnosis were identified by the University of North Carolina (UNC)–Lineberger Comprehensive Cancer Center and the NC Central Cancer Registry Rapid Case Ascertainment Core Facility. Participants were visited in their home or another location of their choice by a trained registered nurse an average of 23.7 weeks after diagnosis (median = 19.7 weeks, range = 6.1–119.6 weeks). Written informed consent and permission for medical record release was obtained. A structured questionnaire was administered by a trained nurse in order to collect information on demographics and PCa risk factors. The nurse then made anthropometric measurements including weight and height. Participants who did not have a urinary catheter in place provided 20 mL urine samples. In this study, we included 463 African-American and 491 European-American men with available urine samples in which arsenical species were measured. 

All PCaP protocols and materials were approved by Institutional Review Boards (IRBs) at UNC Chapel Hill and Louisiana State University Health Sciences Center, by the Department of Defense Human Subjects Research Review Board, and by IRBs at other institutions and hospitals as required.

### 2.2. Outcome Assessment

The main outcome of interest in this study, PCa aggressiveness at diagnosis, was determined from several clinical measures abstracted from medical records, which included clinical Gleason grade and stage, and diagnostic PSA. PCa aggressiveness was categorized as (1) low aggressive (Gleason score < 7 and clinical T categories (cT) cT1 (clinically inapparent tumor that is not palpable) or cT2 (tumor is palpable and confined within prostate), and PSA < 10 ng/mL), versus (2) intermediate or high aggressive (all other cases). Men with low aggressive PCa served as the reference group in all analyses.

### 2.3. Arsenic Assessment

Urinary arsenic measurements were made using an Agilent (Santa Clara, CA, USA) 7500cx inductively-coupled plasma mass spectrometer (ICPMS) from 2015 to 2017 at the University of North Carolina (UNC) Biomarker Mass Spectrometry Core Facility. Helium was flowed through the octopole collision/reaction cell at 4 mL/min to remove argon chloride matrix interferences. An Agilent Bio-Inert Liquid Chromatography (HPLC) system was used for chromatographic separations of arsenic metabolites. Total arsenic measurements followed the methods of Heitland and Köster [23]. A 7-fold dilution of 400 µL urine with 1% nitric acid was quantified against an external calibration curve. Tellurium was used as an internal standard and added through a T-connection. The recovery of quality control (QC) standards (*n* = 61) was 98 ± 12%. Arsenical species were separated on a PRPX-100 (5 µm, 150 × 4.1 mm) anion exchange column (Hamilton Company, Reno, NV, USA) using a gradient elution [24]. Mobile phases A & B consisted of A-ammonium carbonate and TRIS at pH 8.7, and B-ammonium carbonate, TRIS, and ammonium sulfate at pH 8.0. The column eluent was plumbed directly into the ICPMS. Inorganic arsenic injected post-column was used as an internal standard. Standard recoveries (*n* = 37) were Ab = 99 ± 16%, iAs^III^ = 101.6 ± 22%, iAs^V^ = 98 ± 16%, DMA = 99 ± 17%, and MMA = 95 ± 24%. Spike recoveries (*n* = 22) were Ab = 93 ± 9%, iAs^III^ = 95 ± 9%, iAs^V^ = 87 ± 9%, DMA = 92 ± 9%, and MMA = 100 ± 10%. Coefficients of variation (CVs) were total As = 12.2%, Ab = 16.2%, iAs^III^ = 16.3%, iAs^V^ = 21.7%, DMA = 17.2%, and MMA = 25.3%. Limits of detection (LOD) were 0.175 ppb for total arsenic and 0.800 ppb for arsenical species. The proportions of samples <LOD were 0.1% for total As (*n* = 1), 23.9% for Ab (*n* = 228), 59.6% for iAs (*n* = 567), 8.3% for DMA (*n* = 79), and 74.4% for MMA (*n* = 708). Only 118 participants had iAs, MMA, and DMA measurements >LOD, which precluded us from considering arsenic methylation efficiency. For analyses using continuous measures, observations below the LOD were set to the lowest observed value for that arsenical species. Specific gravity (SG), which was used to adjust samples for urine dilution using the formula As value × (mean SG-1)/(individual SG-1) [25], was measured using the Reichert TS400 Total Solids Refractometer (Reichert Inc., Depew, NY, USA). 

### 2.4. Covariate Assessment

Covariates associated with PCa incidence were identified from the PCa epidemiologic literature [9,26]. Potential confounders obtained from the interviewer-administered questionnaire included age (40–54, 55–75, or >75 years), race (European-American or African-American), education (<high school; high school graduate; vocational, technical, some college; or college graduate), marital status (married or unmarried), smoking status (never, former, or current smoker), and residence (urban or rural, based on Census 2010 county classifications [27]). Alcohol intake and seafood consumption in the 12 months prior to diagnosis were assessed using the National Institutes of Health Diet History Questionnaire and estimated using the Diet*Calc Analysis Software [28]. Frequency and amount of intake of beer, wine, and liquor were assessed using 8 items. Alcohol intake was categorized into tertiles using the following cut-points: ≤0.04, 0.05–7.09, or ≥7.10 g. Frequency and amount of consumption of fried fish, unfried shellfish, dark meat fish, and fresh tuna were assed using 8 items. Seafood consumption, which is associated with higher levels of organic arsenic [29], was categorized into tertiles using the following cut-points: ≤0.29, 0.30–0.68, or >0.68 oz. Body mass index (BMI, <25, 25–29.9, 30–39.9, and ≥40 kg/m^2^) was calculated from measured height and weight. Receipt of androgen deprivation therapy or radiotherapy (yes or no) prior to urine sample collection was determined by medical chart abstraction.

### 2.5. Statistical Analysis

Because the distributions of SG-adjusted urinary arsenical species were right skewed, we used non-parametric Wilcoxon signed-rank tests to evaluate the differences in the distributions of Ab-corrected total As (calculated as the average total As over repeated measures minus Ab, hereafter referred to as total As), ΣAs (calculated as the sum of iAs^III^, iAs^V^, DMA, and MMA), and DMA by PCa aggressiveness and by race. We did not evaluate the differences in the distributions of iAs and MMA by PCa aggressiveness given the high proportions of men below the LOD for these arsenical species. In multivariable analyses, we examined tertiles of SG-adjusted total arsenic, DMA, MMA, and ΣAs. For iAs and MMA, men with non-detectable levels were categorized into the lowest exposure group and men with detectable levels were categorized into quantiles at the medians. In the analysis using total arsenic, we excluded 60 men with negative values, which indicated more arsenic from food/fish than from water. In sensitivity analyses, in which we included these 60 men as a separate group, interpretations were not materially different (results not shown). We used multivariable logistic regression to estimate the odds ratios (ORs) and 95% confidence intervals (CIs) for the associations between tertiles or quantiles of total arsenic or arsenical species and PCa aggressiveness. We also examined log-linear trends (*P*_Trend_) using ln-transformed SG-adjusted arsenic levels. Logistic regression models were adjusted for age (i.e., age-adjusted model) and then adjusted for additional covariates including race, education, marital status, smoking status, body mass index, residence, alcohol consumption, and seafood consumption (i.e., multivariable-adjusted). We examined effect measure modification by stratifying the fully-adjusted logistic regression models by race (European-American vs. African-American). We evaluated multiplicative interactions (*P*_Interaction_) using likelihood ratio tests that compared models with interaction terms for categorical arsenical species-by-race interactions against reduced models without the interaction terms. 

All analyses were conducted using SAS version 9.4 (SAS Institute Inc., Cary, NC, USA) and used a critical alpha of 0.05.

## 3. Results

Approximately half (48.5%) of the men included in this study were African-Americans, 34.7% were college graduates, and 75.4% reported being married or living as married (Table 1). The majority of men reported being former (51.5%) or current (15.8%) smokers, and over one-third had a BMI ≥ 30 kg/m^2^. Among men with intermediate or high aggressive PCa, 55.7% were African-American, 18.7% were under the age of 55, and 17.9% reported being current smokers. The mean ages at diagnosis were 63.4 among men with intermediate/high aggressive PCa and 61.9 among men with low aggressive PCa.

SG-adjusted arsenic levels ranged from 0.0158–182 ppb (IQR = 2.20–7.59 ppb) for total As, 0.239–17.1 ppb (IQR = 2.39–7.65 ppb) for iAs, 0.509–62.1 ppb (IQR = 1.91–5.88 ppb) for DMA, and 0.288–2.94 ppb (IQR = 0.288–0.384 ppb) for MMA. Of note, not all arsenical species were selected from the spectrometer results. Therefore, ΣAs was not equal to the total As and ranged from 0.244–64.0 ppb (IQR = 2.32–7.10 ppb). In bivariate analyses, median levels did not differ between men with low aggressive versus intermediate/high aggressive PCa for total arsenic (8.76 vs. 8.67 ppb; *p* = 0.49), ΣAs (3.80 vs. 4.14 ppb; *p* = 0.58), and DMA (3.20 vs. 3.51 ppb; *p* = 0.44). Median total arsenic levels were higher in European-American men than in African-American men (4.51 versus 3.93 ppb; *p* = 0.03), but did not differ by race for ΣAs (4.14 versus 3.87 ppb; *p* = 0.23), iAs (0.239 versus 0.239 ppb; *p* = 0.07), DMA (3.54 versus 3.14 ppb; *p* = 0.57), and MMA (0.289 versus 0.289 ppb; *p* = 0.09).

In the multivariable logistic regression analyses, total arsenic and arsenical species were not strongly associated with PCa aggressiveness (Table 2). The highest (vs. lowest) tertile of total arsenic was associated with an OR for PCa aggressiveness of 1.26 (95% = 0.89–1.80), and a one-ln unit increase in total As was associated with an OR of 1.00 (95%C = 0.88–1.13; *P*_Trend_ = 0.96) in fully-adjusted models. By race, the highest (vs. lowest) tertile of total arsenic was associated with an OR of 1.77 (95% CI = 1.05–2.98) among European-American men, and with an OR of 0.94 (95% CI = 0.57–1.56) among African-American men (*P*_Interaction_ = 0.04) in fully-adjusted models (Table 3). Race did not modify the associations between ΣAs (*P*_Interaction_ = 0.89), iAs (*P*_Interaction_ = 0.79), DMA (*P*_Interaction_ = 0.71), and MMA (*P*_Interaction_ = 0.64) and PCa aggressiveness.

## 4. Discussion

The primary aims of this study were to examine the associations between total arsenic and arsenical species and prostate cancer aggressiveness among African-American and European-American men with PCa from NC, USA. Neither the total urinary arsenic nor the arsenical species were associated with PCa aggressiveness when the entire cohort was considered. However, race-stratified analyses revealed that the highest versus lowest tertile of total urinary arsenic was associated with a 70% increase in the odds of intermediate or aggressive PCa among European-American, but not African-American, men.

Most studies of arsenic and PCa have been ecological and have focused on PCa mortality in countries with water arsenic levels of up to 2500 ppb [11] and have thus largely not included African Americans. However, previous studies have reported consistently strong dose-response associations between high levels of arsenic in drinking water and PCa mortality [30,31,32,33]. Of note, these countries with high exposure to arsenic report lower incidence of prostate cancer than developed countries such as the US [34]; however, this may be due to a selection bias as arsenic is a multisite carcinogen and thus men may die of arsenic-induced cancers such as cancers of the lung, bladder, kidney, or skin before the development of aggressive prostate cancer. Thus, our hypothesis that low-level arsenic exposure is associated with PCa aggressiveness is plausible. In the US, where there is low-level arsenic exposure, at least three ecological studies have examined arsenic in drinking water in association with PCa mortality [35], and one prospective study examined low-to-moderate urinary arsenic levels and risk of PCa mortality [33]. In the study by Lewis et al., elevated PCa mortality rates were reported among the Utah men exposed to drinking water arsenic levels <200 ppb [35]. Given that 90% of the population in Utah is White or European [36], our results among European-American men may be most comparable and are consistent with those by Lewis et al. In the study by Garcia-Esquinas et al., there was a 4-fold increase in the risk of PCa mortality among American-Indian men in highest versus lowest tertiles of total urinary arsenic [33]; however, urinary arsenic levels in their study were higher than those in our study with many more samples with detectable levels.

Growing epidemiologic evidence suggests that iAs may impact PCa risk, but the molecular mechanisms by which arsenic may initiate or promote PCa are not understood well. In vitro approaches including the use of the nontumorigenic human prostate epithelial cell line RWPE-1 have been instrumental for studying the molecular events in iAs-associated carcinogenesis. RWPE-1 cells continuously exposed to 5 μM arsenite have been shown to increase matrix metalloproteinase-9 (MMP-9) secretion (a marker of aggressive malignancies) two-fold compared with control, develop androgen independence, and produce undifferentiated malignant epithelial tumors when inoculated into nude mice [37]. The iAs-induced transformation is hypothesized to be mediated by decreased DNA methyltransferase activity, genomic DNA hypomethylation, and unmutated K-ras oncogene overexpression [38]. Methylation of arsenic facilitates urinary excretion; however, laboratory evidence indicates that methylation may increase the toxicity and tumor-promoting effects of arsenical species [19]. Other hypothesized mechanisms of arsenic carcinogenic action include induction of oxidative damage [39,40], stimulation of proliferation [41], and induction of chromosomal abnormalities [42].

Our study revealed an association between the levels of the arsenical species and PCa aggressiveness among European-American men, but not for African-American men. We propose two potential explanations for this observation. First, biological differences in tumors between African-American and European-American men may make European-American men more susceptible to the effects of arsenic. For example, MMP-9 induction by arsenic, which is expressed at higher concentrations in tumors of African-American than European-American men [43,44] may have minimal additional influence among African-American men. Whereas in European-American men with low basal MMP-9 levels, the As-MMP-9 induction may be sufficient to increase their risk. Second, associations by race may be due to differences in efficiency of arsenic metabolism and clearance. Research on genetic variants in As metabolism pathway genes in African Americans is limited, but a recently published study examined candidate variants in arsenic (III) methyltransferase (*AS3MT*), which catalyzes conversion of iAs to methylated products. Their study was underpowered to examine differences by race (*n* = 84 non-Hispanic whites and 56 African Americans), but slightly higher minor allele frequencies were reported in African-Americans versus non-Hispanic Whites for the three *AS3MT* single nucleotide polymorphisms [45]. Additional research aimed at elucidating the biological mechanisms underlying these associations may be warranted if our findings reported here are replicated in more methodologically rigorous studies.

Our study should be interpreted in light of its strengths and limitations. While an evaluation of well-water arsenic exposure and PCa aggressiveness was not the original aim of the PCaP study, our invaluable resource of well-characterized men with incident PCa, living in NC, a region with high well water usage and known private well As contamination, along with a large biobank of urine specimens collected near the time of PCa diagnosis, seemed an appropriate data set with which to explore arsenic exposure in association with PCa. This study included a large sample of men with equal proportions of African-American and European-American men, and used a urinary biomarker measure of arsenic exposure, a comprehensive measurement of arsenical species, and a robust clinical definition of PCa aggressiveness. This study, however, was limited by the cross-sectional design, which limits the ability to make causal inferences. We measured arsenic in urine samples obtained after PCa diagnosis and so it is unclear whether this exposure reflects levels during the relevant etiologic time window of PCa development. Nonetheless, our results may still be relevant for understanding disease progression and the cross-sectional design is an improvement on previous ecological studies as we were able to control for individual-level confounding factors and examine differences by race. Although we relied on a single measurement of urinary arsenic, total urinary arsenic shows fair to good reproducibility over years, with reported intraclass correlations ranging from 0.66 to 78 [46]. Absorbed arsenic has a biological half-life of four days [20]; therefore, urinary measurements reflect recent exposures. However, ongoing steady exposure, as would be expected with exposure through drinking water, should allow for a measured level to reflect chronic long-term exposure. We assumed this to be the case in this population of NC men, since arsenic exposure from contaminated private domestic wells in NC, especially along the Carolina terrane, is likely to remain constant over time [16]. In this study, there were high proportions of men with arsenical species levels below the limits of detection and high CVs due to the low levels of some arsenical species (though these CVs are consistent with other studies [47,48]). This also precluded us from exploring arsenic methylation efficiency, which is of established importance in the underlying susceptibility to arsenic toxicity. Thus, our interpretations for the arsenical species may require caution. Last, although we considered many important potential confounders, we did not collect information on the participants’ home water source or duration at that address, consumption of bottled water or tap water, or consumption of filtered water, and we did not measure arsenic levels in their home drinking water. However, prior studies in North Carolina indicate that exposure to arsenic from contaminated private domestic wells is a public health concern [16].

## 5. Conclusions

More than two million people in NC drink water from unmonitored private wells [17], which have been found to be contaminated with arsenic levels ranging from 1 to 800 μg/L [16]. Exposure to arsenic as measured in urine was low in this cohort, but these levels may still negatively impact the health of men residing in NC. Our finding of an increase in the odds of intermediate or aggressive PCa among European-American men, but not among African-American men, requires further investigation as PCa is a leading cause of morbidity and mortality among US men. 

## Figures and Tables

**Table 1:** Distribution of select characteristics among the Prostate Cancer Project (PCaP) men with available estimates of urinary arsenic overall and by quantiles of specific gravity-adjusted total arsenic (*n* = 954).

	Overall	Prostate Cancer Aggressiveness	
		Low Aggressive	Intermediate/High Aggressive
	*n* (%)	*n* (%)	*n* (%)
Race			
African-American	463 (48.5)	204 (41.7)	259 (55.7)
European-American	491 (51.5)	285 (58.3)	206 (44.3)
Age at diagnosis, years			
<55	197 (20.6)	110 (22.5)	87 (18.7)
55–75	699 (73.3)	360 (73.6)	339 (72.9)
>75	58 (6.1)	19 (3.9)	39 (8.4)
Education			
<High school	162 (17.0)	65 (13.3)	97 (20.9)
High school graduate	238 (25.0)	126 (25.8)	112 (24.1)
Vocational, technical, some college	222 (23.3)	113 (23.1)	109 (23.5)
College graduate	331 (34.7)	185 (37.8)	146 (31.5)
Missing	1	0	1
Marital status			
Unmarried	234 (24.6)	106 (21.7)	128 (27.6)
Married	719 (75.4)	383 (78.3)	336 (72.4)
Missing	1	0	1
Smoking status			
Never smoker	312 (32.8)	177 (36.3)	135 (29.1)
Former smoker	490 (51.5)	244 (50.0)	246 (53.0)
Current smoker	150 (15.8)	67 (13.7)	83 (17.9)
Missing	2	1	1
Body mass index, kg/m^2^			
<25	173 (18.3)	90 (18.5)	83 (18.1)
25–29.9	403 (42.6)	217 (44.6)	186 (40.5)
30–39.9	328 (34.7)	164 (33.7)	164 (35.3)
≥40	42 (4.4)	16 (3.3)	26 (5.7)
Missing	8	2	6
Residence			
Urban	719 (75.4)	376 (76.9)	343 (73.8)
Rural	235 (24.6)	113 (23.1)	122 (26.2)
Alcohol consumption (g)			
≤0.04	335 (35.1)	172 (35.2)	163 (35.1)
0.05–7.09	299 (31.3)	148 (30.3)	151 (32.5)
≥7.10	320 (33.5)	169 (34.6)	151 (32.5)
Seafood consumption (oz)			
≤0.29	323 (33.9)	166 (33.9)	157 (33.8)
0.30–0.68	315 (33.0)	161 (32.9)	154 (33.1)
>0.68	316 (33.1)	162 (33.1)	154 (33.1)
Receipt of androgen deprivation therapy or radiotherapy prior to urine sample collection			
No	765 (80.2)	448 (91.6)	317 (68.2)
Yes	189 (19.8)	41 (8.4)	148 (31.8)

**Table 2:** Odds ratios (ORs) and corresponding 95% confidence intervals (CIs) for the associations between quantiles of specific gravity-adjusted urinary arsenic concentrations and prostate cancer aggressiveness among PCaP men, (*n* = 954).

Arsenical Species, ppb ^a^	Median, ppb	Intermediate/High vs.Low Aggressive	Age-Adjusted		Multivariable-Adjusted	
			OR (95% CI) ^b^	*P*_Trend_^c^	OR (95% CI) ^d^	*P*_Trend_^c^
Total As						
≤2.83	1.56	140/156	1.00		1.00	
2.83–6.02	6.01	135/161	0.93 (0.67–1.29)		1.02 (0.72–1.44)	
≥6.03	10.01	155/143	1.23 (0.89–1.70)		1.26 (0.89–1.80)	
Ln(Total As)			1.01 (0.90–0.13)	0.85	1.00 (0.88–1.13)	0.96
ΣiAs ^e^						
<2.82	1.71	159/158	1.00		1.00	
2.82–5.71	3.97	145/170	0.85 (0.62–1.17)		0.89 (0.64–1.25)	
≥5.72	9.19	159/159	1.02 (0.75–1.40)		0.97 (0.69–1.37)	
Ln(ΣAs)			1.03 (0.92–1.17)	0.59	1.03 (0.90–1.17)	0.72
iAs ^f^						
<LOD	<LOD	289/277	1.00		1.00	
≥LOD–0.922	0.619	84/107	0.76 (0.54–1.06)		0.76 (0.54–1.09)	
≥0.923	1.74	90/103	0.86 (0.62–1.19)		0.84 (0.59–1.19)	
Ln(iAs)			0.96 (0.84–1.11)	0.59	0.97 (0.84–1.12)	0.67
DMA						
<2.34	1.41	150/167	1.00		1.00	
2.34–4.73	3.30	155/160	1.08 (0.79–1.48)		1.16 (0.83–1.63)	
≥2.74	7.59	158/160	1.12 (0.82–1.53)		1.09 (0.77–1.55)	
Ln(DMA)			1.06 (0.92–1.21)	0.42	1.04 (0.89–1.21)	0.65
MMA						
<LOD	<LOD	360/346	1.00		1.00	
≥LOD–0.759	0.566	52/70	0.74 (0.50–1.09)		0.85 (0.56–1.28)	
≥7.60	1.05	51/71	0.73 (0.49–1.08)		0.82 (0.54–1.24)	
Ln(MMA)			0.80 (0.62–1.04)	0.09	0.90 (0.68–1.18)	0.44

As, arsenic; DMA, dimethyl arsenic; LOD, limit of detection; MMA, monomethyl arsenic; PPB, parts per billion; SG, specific gravity. ^a^ Adjusted for urine SG = arsenic value x (mean SG − 1)/(individual SG − 1). ^b^ Adjusted for age (40–54, 55–75, or 76–69 years). ^c^ *P*_Trend_ based on ln-transformed specific gravity-adjusted arsenic levels. ^d^ Adjusted for age (40–54, 55–75, or 76–69 years); race (European-American or African-American); education (<High school, High school graduate, Vocational/technical/some college, or College graduate); marital status (Married or Unmarried); smoking status (Never, Former, or Current smoker); body mass index (<25, 25–29.9, 30–39.9, or ≥40 kg/m^2^); residence (Urban or Rural); alcohol consumption (≤0.04, 0.05–7.09, or ≥7.10 g); seafood consumption (≤0.29, 0.30–0.68, or >0.68 oz); and receipt of androgen deprivation therapy or radiation therapy prior to urine sample collection (no, yes). ^e^ ΣAs calculated as iAs^III^+iAs^V^+DMA+MMA. ^f^ iAs = iAs^III^+iAs^V^.

**Table 3:** Race-stratified odds ratios (ORs) and corresponding 95% confidence intervals (CIs) for the associations between quantiles of specific gravity-adjusted urinary arsenic concentrations and prostate cancer aggressiveness among PCaP men, (*n* = 954).

Arsenical Species, ppb ^a^	African-American (*n* = 463)				European-American (*n* = 491)				*P*_Interaction_^d^
	Median, ppb	Intermediate/High vs. Low Aggressive	OR (95% CI) ^b^	*P*_Trend_^c^	Median, ppb	Intermediate/High vs. Low Aggressive	OR (95% CI) ^b^	*P*_Trend_^c^	
Total As									0.04
≤2.83	1.59	79/61	1.00		1.47	61/95	1.00		
2.83–6.02	4.37	79/57	1.16 (0.69–1.94)		4.12	56/104	0.92 (0.56–1.50)		
≥6.03	10.16	85/75	0.94 (0.57–1.56)		9.70	70/68	1.77 (1.05–2.98)		
Ln(Total As)			0.95 (0.79–1.13)	0.55			1.04 (0.86–1.25)	0.68	
ΣAs^e^									0.89
<2.82	1.61	85/65	1.00		1.81	74/93	1.00		
2.82–5.71	3.95	78/64	0.99 (0.60–1.63)		3.97	67/106	0.79 (0.49–1.26)		
≥5.72	9.29	95/74	1.00 (0.61–1.63)		9.12	64/85	0.91 (0.55–1.52)		
Ln(ΣAs)			1.03 (0.86–1.25)	0.73			0.99 (0.81–1.21)	0.93	
iAs ^f^									0.79
<LOD	<LOD	156/112	1.00		<LOD	133/165	1.00		
≥LOD–0.922	0.593	46/49	0.74 (0.45–1.22)		0.638	38/58	0.76 (0.46–1.26)		
≥0.923	1.70	56/42	0.90 (0.54–1.49)		1.84	34/61	0.76 (0.46–1.25)		
Ln(iAs)			1.05 (0.84–1.30)	0.69			0.89 (0.72–1.10)	0.27	
DMA									0.71
<2.34	1.38	79/68	1.00		1.50	71/99	1.00		
2.34–4.73	3.38	87/59	1.34 (0.81–2.22)		3.22	68/101	0.97 (0.60–1.55)		
≥2.74	7.67	92/76	1.11 (0.68–1.83)		7.54	66/84	1.02 (0.61–1.70)		
Ln(DMA)			1.02 (0.82–1.26)	0.86			1.03 (0.82–1.30)	0.80	
MMA									0.64
<LOD	<LOD	207/146	1.00		<LOD	153/200	1.00		
≥LOD–0.759	0.544	25/28	0.69 (0.37–1.28)		0.574	27/42	0.97 (0.56–1.69)		
≥7.60	0.994	26/29	0.76 (0.41–1.42)		1.13	25/42	0.89 (0.49–1.62)		
Ln(MMA)			0.88 (0.58–1.34)	0.55			0.91 (0.62–1.33)	0.63	

As, arsenic; DMA, dimethyl arsenic; LOD, limit of detection; MMA, monomethyl arsenic; PPB, parts per billion; SG, specific gravity. ^a^ Adjusted for urine SG = arsenic value x (mean SG − 1)/(individual SG − 1). ^b^ Adjusted for age (40–54, 55–75, or 76–69 years); education (<High school, High school graduate, Vocational/technical/some college, or College graduate); marital status (Married or Unmarried); smoking status (Never, Former, or Current smoker); body mass index (<25, 25–29.9, 30–39.9, or ≥40 kg/m^2^); residence (Urban or Rural); alcohol consumption (≤0.04, 0.05–7.09, or ≥7.10 g); and seafood consumption (≤0.29, 0.30–0.68, or >0.68 oz); and receipt of androgen deprivation therapy or radiation therapy prior to urine sample collection (no, yes). ^c^ *P*_Trend_ based on ln-transformed specific gravity-adjusted arsenic levels. ^d^ *P*_Interaction_ derived from categorical arsenic quantile-by-race interactions in the logistic regression models. ^e^ ΣAs calculated as iAs^III^+iAs^V^+DMA+MMA. ^f^ iAs = iAs^III^+iAs^V^.
